# Alterations in mitochondrial morphology as a key driver of immunity and host defence

**DOI:** 10.15252/embr.202153086

**Published:** 2021-08-02

**Authors:** Mariana P Cervantes‐Silva, Shannon L Cox, Annie M Curtis

**Affiliations:** ^1^ School of Pharmacy and Biomedical Sciences and Tissue Engineering Research Group Royal College of Surgeons in Ireland Dublin Ireland

**Keywords:** mitochondrial dynamics, immune response, bacteria, virus, therapy, Immunology, Microbiology, Virology & Host Pathogen Interaction, Organelles

## Abstract

Mitochondria are dynamic organelles whose architecture changes depending on the cell’s energy requirements and other signalling events. These structural changes are collectively known as mitochondrial dynamics. Mitochondrial dynamics are crucial for cellular functions such as differentiation, energy production and cell death. Importantly, it has become clear in recent years that mitochondrial dynamics are a critical control point for immune cell function. Mitochondrial remodelling allows quiescent immune cells to rapidly change their metabolism and become activated, producing mediators, such as cytokines, chemokines and even metabolites to execute an effective immune response. The importance of mitochondrial dynamics in immunity is evident, as numerous pathogens have evolved mechanisms to manipulate host cell mitochondrial remodelling in order to promote their own survival. In this review, we comprehensively address the roles of mitochondrial dynamics in immune cell function, along with modulation of host cell mitochondrial morphology during viral and bacterial infections to facilitate either pathogen survival or host immunity. We also speculate on what the future may hold in terms of therapies targeting mitochondrial morphology for bacterial and viral control.

## Graphical abstract

Mitochondrial dynamics is crucial for several cellular processes including immune cell function. This review discusses how modulation of host cell mitochondrial morphology affects pathogen survival and host immunity and speculates on related therapeutic approaches.

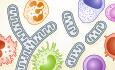

**Glossary**
6‐OHDA: 6‐hydroxydopamine
APC: Antigen Presenting Cell
ASC: Apoptosis‐associated speck‐like
ATP: Adenosine triphosphate
BCR: B‐cell antigen receptor
BMDMs: Bone marrow‐derived macrophages
BNIP3L: BCL2 Interacting Protein 3 Like
cAMP: Cyclic adenosine monophosphate
CARD: Caspase activation and recruitment domains
CCCP: Carbonyl cyanide m‐chlorophenylhydrazone
CMT2A: Charcot‐Marie‐Tooth disease type 2A
CoV: Coronaviruses
CpG: Cytosine‐phosphorothioate‐guanine
DAMPs: Damage‐associated molecular patterns
DCs: Dendritic cells
DENV: Dengue virus
DRP1: Dynamin‐related protein 1
ER: Endoplasmic reticulum
ETC: Electron transport chain
FAO: Fatty acid oxidation
Fis1: Mitochondrial Fission protein 1
fMLP: N‐Formylmethionyl‐leucyl‐phenylalanine
FUNDC1: FUN14 domain‐containing protein 1
GM‐CSF: Granulocyte‐macrophage colony‐stimulating factor
GTP: Guanosine‐5'‐triphosphate
HBV: Hepatitis B Virus
HCC: Hepatocellular carcinoma
HCMV: HIV‐1/human cytomegalovirus
HCV: Hepatitis C Virus
HIV‐1: Human immunodeficiency virus
hMDMs: Human monocyte‐derived macrophages
IAV: Influenza A virus
IFN: Interferon
Ig: Immunoglobulin
IKK: IκB kinase
IL: Interleukin
IMM: Inner mitochondrial membrane
IMS: Intermembrane space
INF2: Inverted Formin 2
IRF: Interferon regulatory factor
IS: Immune synapse
LC3: light chain 3
LLO: Listeriolysin O
LprG: Mycobacterium tuberculosis lipoprotein
LPS: Lipopolysaccharide
MAVS: Mitochondrial anti‐viral signalling protein
MCU: Mitochondrial calcium uniporter
MDA‐5: Melanoma differentiation‐associated protein 5
mDCs: Myeloid DCs
MEFs: Mouse embryonic fibroblasts
MFF: Mitochondrial Fission Factor
MFN: Mitochondrial fusion proteins
MiD: Mitochondrial dynamics protein
MTFP1: Mitochondrial Fission Process 1
mtROS: Mitochondrial reactive oxygen species
NADPH: Nicotinamide adenine dinucleotide phosphate
NET: Neutrophil extracellular trap
NF‐κB: Nuclear factor‐kappa B
NIX: NIP3‐like protein X
NK: Natural Killer
NLRP3: NLR family pyrin domain‐containing 3
NOD2: Nucleotide‐binding oligomerisation domain‐containing protein 2
OMM: Outer mitochondrial membrane
OPA: Optic atrophy gene 1
ORF: Open reading frame
OxPhos: Oxidative phosphorylation
PAMPs: Pathogen‐associated molecular patterns
pDCs: Plasmacytoid DCs
PHA: pulmonary arterial hypertension
PINK1: PTEN‐induced kinase 1
PKA: Protein kinase A
PRR: Pathogen recognition receptor
PRRs: Pattern recognition receptors
PTMs: Post‐translational modifications
RIG‐I: Retinoic acid‐inducible gene I
RLRs: (RIG‐I)‐like receptors
RNA: Ribonucleic acid
SARS: Severe acute respiratory syndrome
Spire1c: Spire type actin nucleation factor 1

## Introduction: importance of mitochondria in immunity

The immune system is crucial for host survival and defence against foreign invaders such as bacteria, viruses and parasites. This system is an ensemble of specialised organs, cells and molecules, which sense and protect the host from pathogen infection (Delves & Roitt, 2000). The innate immune response is the non‐specific, rapid first line of defence against infection and involves the recruitment and activation of granulocytes, monocytes, macrophages, dendritic cells and natural killer cells. The adaptive immune response is the highly specific second line of defence, and a slower and long‐lasting response consisting of T and B cells (Parkin & Cohen, 2001). Together these cells recognise the foreign agent and mount an inflammatory response, which, if successful, results in elimination of the pathogen, resolution of inflammation, return to tissue homeostasis and a memory of the pathogen should reinfection occur (Ayala *et al*, 2008). What has become apparent over the last decade is that each immune cell has different metabolic requirements directing their effector function during discrete stages of the immune response against pathogens (O’Neill *et al*, 2016). This has given rise to a new field called immunometabolism. Due to this emerging field, we now appreciate the critical role which mitochondria play in modulating the immune response and this is the major topic of this review.

Ask any undergraduate student what mitochondria do, and you will get the same response delivered with gusto “*mitochondria are the powerhouse of the cell*”. Certainly, mitochondria through the electron transport chain (ETC) are uniquely specialised to meet the cell’s energy requirement by producing adenosine triphosphate (ATP) via oxidative phosphorylation (OxPhos). Nonetheless, it is now unequivocally clear that mitochondria play a multitude of cellular functions beyond energy production (McBride *et al*, 2006). We now appreciate that mitochondria are essential organelles directing the fate and function of innate and adaptive immune cells (Angajala *et al*, 2018). Mitochondria are vital for modulating immune cell activation, differentiation, metabolism, effector functions and immune survival (Rambold & Pearce, 2018). Therefore, it makes sense that, upon invasion, numerous pathogens would target mitochondria in an attempt by the pathogen to manipulate this organelle for its own advantage.

Mitochondria are double‐membrane bound organelles consisting of an outer membrane (OMM) and an inner membrane (IMM), with an intermembrane space (IMS) in between. Multiple proteins reside in the OMM including mitochondrial anti‐viral signalling protein (MAVS) (Horner *et al*, 2011), mitochondrial fusion proteins MFN1 and MFN2 (Chen *et al*, 2003), anti‐apoptotic proteins and mitochondrial voltage‐dependent anion channel (VDAC). Changes in OMM permeability allows the OMM to act as a signalling platform between the cytosol and mitochondria, with VDAC facilitating the transport of ions and small molecules (De Stefani *et al*, 2012). The IMM contains the fusion protein OPA1, as well as the electron transport chain (ETC), which facilitates the generation of ATP via OxPhos (Rambold & Pearce, 2018). The ETC is also the main producer of reactive oxygen species (mtROS), which is crucial for anti‐pathogenic responses by directly killing the pathogen, or mtROS can activate other pathways such as pathogen recognition receptor (PRR) activation and neutrophil extracellular trap (NET) formation (Pinegin *et al*, 2018).

Mitochondria are also intrinsically involved in cell death processes such as necroptosis, apoptosis and pyroptosis. Pyroptosis relies on the activation of inflammasomes via mtROS release into the cytosol, a mechanism necessary for the production of pro‐inflammatory mediators (Wang *et al*, 2019).

Mitochondrial dynamics as we will describe below in more detail plays a crucial role in mitochondrial function. Pathogens have evolved mechanisms to manipulate mitochondrial dynamics, leading to mitochondrial dysfunction and persistent infection. In this review, we will first discuss the mechanisms driving mitochondrial remodelling. Next, we discuss how mitochondrial dynamics directs immune cell function through the process of inflammation. Then, we review how pathogens or the host alters the mitochondrial architecture in infected cells in the battle between pathogen and host. We discuss what therapeutic opportunities may exist in exploiting mitochondrial dynamics for anti‐microbial and anti‐viral control. Finally, we conclude on many of the outstanding questions which exist in this field and what the future might hold for mitochondrial dynamics in immunity.

## The mechanics of mitochondrial morphology

Mitochondrial dynamics refers to the dynamic remodelling of the mitochondrial network with regard to size, shape, activity, trafficking and connectivity of this organelle. Mitochondrial dynamics is determined by the equilibrium of fission versus fusion. Although arbitrary, fission is normally characterised by punctuated mitochondria (less than 1 µm in length), and fusion characterised by interlocking connected networks of mitochondria (greater than 3 µm in length) (Park *et al*, 2013; Collins *et al*, 2021; Dowling *et al*, 2021). We will first address the constituents and molecular pathways which dictate the process of mitochondrial fusion and fission. Following that we will then discuss the role of mitochondrial fusion and fission in immune cell metabolism and the response to infection.

### Molecular machinery driving fission and fusion of mitochondria

While mitochondrial dynamics was first observed over 100 years (Lewis & Lewis, 1914), the proteins dictating mitochondrial morphology have been identified and extensively studied in the past 20+ years. Clearly, as the name suggests, mitochondrial dynamics is a highly dynamic process, with changes to mitochondrial morphology reflecting the cell’s adaptation to its surrounding environment. Mitochondrial morphology is a spectrum, with fission and fusion occupying extremities of the morphological spectrum (Galloway *et al*, 2012). Thus, depending on the extracellular conditions, cells will lean to either fusion or fission morphology (Zemirli *et al*, 2018). In order to do so, cells employ an intricate network of proteins, which act to increase one morphological state while inhibiting the other.

### The process of mitochondrial fusion

**Figure 1:**
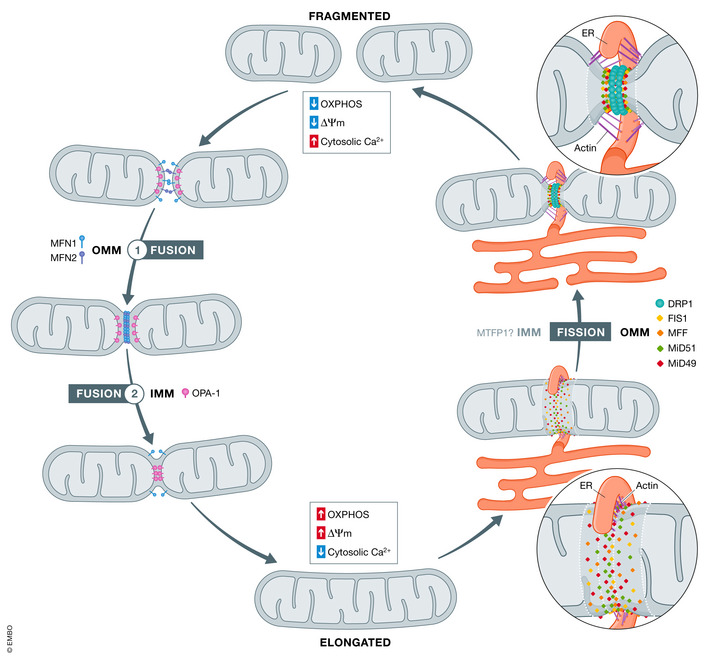
Mitochondrial fusion and fission Mitochondria are constantly changing their morphology depending on the cell's metabolic requirements. Mitochondrial fusion occurs in 2 stages: first, (1) fusion of the OMM occurs after the interaction of MFN1 and MFN2 which allows for increased surface area contact between the two mitochondria. (2) Fusion of the IMM occurs almost simultaneously and is mediated by the GTPase OPA1 that is anchored to the IMM. Mitochondrial fission involves the division of a single mitochondrion into two separate daughter mitochondria. Mitochondrial fission involves contact between the endoplasmic reticulum (ER) and the mitochondria mediated by actin cytoskeletal proteins which carry out constriction of the mitochondria. Once constriction occurs, DRP1 is recruited to fission sites at the mitochondria. DRP1 recruitment is regulated by FIS1, MFF, MiD51 and MiD49, which are adaptor proteins located on the OMM. Source data are available online for this figure.

Fusion involves the fusion of the OMM and IMM resulting in the joining of two mitochondria into one. OMM fusion occurs first and is then swiftly followed by IMM fusion (Fig 1). MFN1 and MFN2 mediate fusion of the OMM through homo‐ and heterotypic interactions powered by guanosine‐5'‐triphosphate (GTP) hydrolysis. Briefly, fusion of the OMM occurs in three steps: (1) OMM tethering between two mitochondria mediated by the MFNs, (2) interaction of MFN1 and MFN2 with their C‐termini to allow for tethering of neighbouring mitochondria in trans leading to increased surface area contact between the two mitochondria (Brandt *et al*, 2016) and (3) GTP hydrolysis‐mediated conformational changes leading to OMM fusion (Ishihara *et al*, 2004).

Fusion of the IMM occurs almost simultaneously with fusion of the OMM and is mediated by the GTPase OPA1 (Sesaki *et al*, 2003). OPA1 is anchored to the IMM by its N‐terminal, and upon fusion of the OMM, OPA1 is cleaved into two forms: a long (L) form and a short (S) form. This proteolytic cleavage is carried out by metalloproteases YME1L and OMA1 in the mitochondria intermembrane space (IMS) (Song *et al*, 2007; Ehses *et al*, 2009). Cleavage of OPA1 generates numerous protein isoforms consisting of higher molecular weight membrane‐anchored L‐forms (L‐OPA1) and non‐membrane‐anchored S‐forms (S‐OPA1) (Wang *et al*, 2021). The individual roles of OPA1 isoforms are reviewed in detail elsewhere (Del Dotto *et al*, 2018). Fusion of mitochondria in a cell is associated with increased OxPhos and ATP production and is characterised by elongated mitochondria (Mishra & Chan, 2016).

### The process of mitochondrial fission

Whereas fusion produces networks of interconnected mitochondria, the process of fission then breaks up that network into distinct fragments Mitochondrial fission involves the division of a single mitochondrion into two separate daughter mitochondria. Similar to fusion, fission occurs at both OMM and IMM, with OMM fission mediated by the cytosolic GTPase DRP1 (Fig 1) (Bleazard *et al*, 1999; Labrousse *et al*, 1999). Initiation of mitochondrial fission involves contact between the endoplasmic reticulum (ER) and the mitochondria mediated by actin cytoskeletal proteins such as spire‐type actin nucleation factor 1 (Spire1c) and inverted formin 2 (INF2) (Friedman *et al*, 2011; Korobova *et al*, 2013; Manor *et al*, 2015). These proteins carry out constriction of the mitochondria. Once constriction occurs, dynamin‐related protein 1 (DRP1) is recruited to fission sites at the mitochondria. DRP1 recruitment is regulated by fission protein 1 (FIS1), mitochondrial fission factor (MFF), MiD51 and MiD49 which are adaptor proteins located on the OMM (Osellame *et al*, 2016). Moreover, DRP1 activity is also regulated by signalling events. For example, mitochondrial fission occurs when DRP1 is dephosphorylated by the phosphatase calcineurin at Ser637, a process which is regulated by calcium content and will be discussed later on (Cereghetti *et al*, 2008). DRP1, upon recruitment, forms oligomeric rings, which promotes mitochondrial scission by GTPase hydrolysis‐mediated conformational changes that constricts the rings (Mears *et al*, 2011).

Although mechanisms surrounding OMM fission are well documented, mechanisms driving IMM fission are less understood. The IMM protein mitochondrial fission process 1 (MTFP1) appears to mediate IMM fission. Overexpression of MTFP1 increases mitochondrial fission, while its loss leads to mitochondrial fusion (Tondera *et al*, 2005). Moreover, Morita *et al* found that MTFP1 is a modulator of DRP1 phosphorylation, the significance of which is detailed below (Morita *et al*, 2017). Other proposed mechanisms of IMM fission are reviewed in detail elsewhere (Tilokani *et al*, 2018).

### Mitophagy, the process of mitochondrial clearance

Mitochondrial autophagy, known as mitophagy, is necessary for the clearance of dysfunctional mitochondria and is regulated by fission and fusion. Clearance of damaged mitochondria is necessary for maintaining the quality of mitochondria and avoids cell apoptosis (Kubli & Gustafsson, 2012). Ubiquitin‐dependent mitophagy is controlled by the PINK1/parkin pathway. PINK1 which is a serine/threonine protein kinase accumulates in the OMM resulting in parkin recruitment to the mitochondria (Lazarou *et al*, 2015). This process is promoted by fission and inhibited by fusion (Twig *et al*, 2008). Parkin recruitment initiates degradation of OMM proteins via ubiquitination, as parkin is a component of a multiprotein E3 ubiquitin ligase complex (Kane *et al*, 2014; Kazlauskaite *et al*, 2014; Koyano *et al*, 2014). This triggers formation of autophagosomes to remove damaged mitochondria from the cell (Rodriguez‐Enriquez *et al*, 2006). Other mechanisms of mitophagy include receptor‐mediated mitophagy. This involves the localisation of the mitophagy receptors BNIP3, NIX and FUNDC1 to the OMM where they interact with LC3 to mediate mitochondrial clearance. These mitophagy receptors ultimately promote fission and subsequent clearance of damaged mitochondria by releasing OPA1 and recruiting DRP1 to the mitochondrial surface (Palikaras *et al*, 2018). As we will see later on, mitophagy is a crucial process for many immune cell functions and implicated in pathogen clearance.

### Cellular events regulating fusion and fission of mitochondria

Mitochondrial fission and fusion are rapid processes dependent on the coordination of numerous proteins in response to various stimuli, such as nutrient availability including glucose levels. For example, in diabetes studies, sustained exposure of heart myoblast cells to high glucose conditions results in fragmented mitochondria (Yu *et al*, 2008). Thus, transcriptional regulation of mitochondrial dynamic proteins is too slow. As such, most of the regulation of mitochondrial remodelling occurs through post‐translational modifications (PTMs) (Tilokani *et al*, 2018). PTMs are key regulators of mitochondrial proteins, which allow for swift and efficient switching to fission and fusion in response to physiological demands. PTMs such as phosphorylation, acetylation, SUMOylation, O‐GlcNAcylation and ubiquitination differentially regulate mitochondrial proteins and the mitochondrial network to meet the cell’s requirements. Reversible phosphorylation of mitochondrial proteins in particular is a well‐documented regulatory mechanism of mitochondrial dynamics. The phosphorylation status of DRP1 on serine residues including serine 616 and serine 637 determines mitochondrial morphology (Archer, 2013).

Mitochondria control cellular localisation and levels of calcium. Many of the metabolic enzymes and proteins within the ETC and the tricarboxylic acid cycle (TCA) are calcium‐dependent and are reviewed elsewhere (Rizzuto *et al*, 2012). Mitochondria sense and take up calcium via the mitochondrial calcium uniporter and release calcium into the cytosol via efflux channels (Kirichok *et al*, 2004). The changes in calcium levels and links to mitochondrial dynamics are complex. Increased cytosolic Ca^2+^ activates the phosphatase calcineurin which dephosphorylates DRP1 at Ser637, leading to mitochondrial fission, promotion of mitophagy and sometimes apoptosis of the cell (Cereghetti *et al*, 2008). On the other hand, Ca^2+^ influx into mitochondria has also been associated with fission of the IMM via OPA1 processing to S‐OPA1 (Cho *et al*, 2017), and increased Ca^2+^ influx is reported to inhibit fusion by inhibiting the oligomerisation of MFN1 (Ishihara *et al*, 2017).

Mitochondria can sense the second messenger cyclic adenosine monophosphate (cAMP) signalling at the OMM, and this is a major regulator of mitochondrial fission and fusion. cAMP activates protein kinase A (PKA), which phosphorylates DRP1 at Ser656, leaving DRP1 inactive in the cytosol and maintaining fusion (Cribbs & Strack, 2007). Activation of PKA is also associated with increased OxPhos and cell survival (Ould Amer & Hebert‐Chatelain, 2018).

There are many more regulatory mechanisms of fission and fusion, allowing mitochondria to function correctly and efficiently. This is especially important in immune cells, as mitochondrial dynamics affect numerous aspects of the immune response to infection. As we will see below, mitochondrial remodelling affects immune cell metabolism, activation, differentiation and cytokine production.

## The role of mitochondrial dynamics in host immunity

The host immune response to infection is an orchestrated response mediated by the temporal coordination of specific immune cells. Starting with pathogen sensing, an inflammatory response is mediated by chemokine and cytokine production, along with increased immune cell recruitment, pathogen clearance and finally resolution. Immune cells have different metabolic requirements during each stage of the host immune response and mitochondria play a critical role in modulating the appropriate response.

### Mitochondrial dynamics in neutrophils

**Figure 2:**
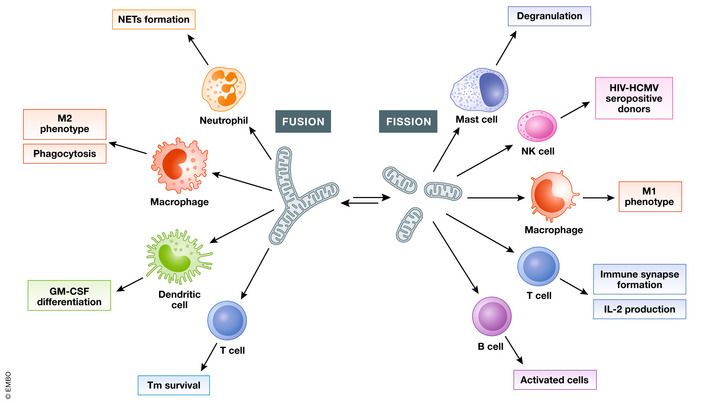
The role of mitochondrial dynamics in host immunity Mitochondria dynamics in immune cells have a crucial role in many aspects of immune function. In neutrophils, the formation of NETs is dependent of mitochondrial fusion. Fusion in macrophages precedes increased phagocytic and bactericidal activity, while fusion in BMDMs is known to promote M2 polarisation. Mitochondrial fusion is also induced during GM‐CSF‐stimulated differentiation of bone marrow progenitor cells to immature dendritic cells. In T memory cells, mitochondrial fusion is essential for cell survival. On the other hand, mitochondrial fission precedes degranulation in the mast cell immune response. NK cells from HIV/HCMS seropositive donors display fragmented mitochondria. BMDMs stimulated with LPS as a model of M1 polarisation display mitochondrial fragmentation. In T cells, IL‐2 production and immune synapse formation are dependent on mitochondrial fission. Finally, mitochondrial fission occurs during B‐cell activation, while naïve B cells have predominantly elongated mitochondria.

One of the first events in the immune response is the recruitment of neutrophils to the site of infection by chemo‐attractants (Parkin & Cohen, 2001). Upon reaching the site of infection, neutrophils employ various mechanisms in order to eliminate pathogens. These mechanisms include phagocytosis, production of mtROS, secretion of granules and the release of pro‐inflammatory cytokines and NETS, all of which promotes pathogenic clearance (Mayadas *et al*, 2014). Neutrophils remodel their mitochondria depending on their activation stage. Nascent neutrophils exhibit tubular mitochondria while non‐surviving neutrophils exhibit an “aggregation” of mitochondria, which is presumed to drive apoptosis (Maianski *et al*, 2002). The formation of NETs is dependent on mitochondrial fusion, as deletion of OPA in neutrophils, decreased ATP levels which is required for microtubule network assembly and NET formation (Fig 2). OPA deletion in mice reduced anti‐bacterial defence against *Pseudomonas aeruginosa* (Amini *et al*, 2018). MFN2, through suppression of Rac activation, controls neutrophil adhesive migration, and MFN2 deletion results in a significant reduction of neutrophil infiltration to the inflamed peritoneal cavity in mice (Zhou *et al*, 2021). Calcium levels are crucial for neutrophil polarisation and chemotaxis (Immler *et al*, 2018). Neutrophil polarisation using N‐formylmethionyl‐leucyl‐phenylalanine (fMLP), a prototypic neutrophil chemotactic factor, induced mitochondrial fission in neutrophils via increased phosphorylation of DRP1 (Ser616). Mitochondrial calcium uniporter (MCU) complex activity was shown to be necessary for this increased fission and DRP1 phosphorylation (Ser616), as inhibition of MCU downregulated DRP1 (Ser616) (Zheng *et al*, 2017).

### Mitochondrial dynamics in mast cells

Mast cells are tissue‐resident immune cells which have high expression of IgE receptor (FcɛRI) and are involved in pathogenic clearance as well as allergic diseases. The presence of IgE and environmental triggers such as neuropeptides leads to degranulation and release of histamine and pro‐inflammatory cytokines such as tumour necrosis factor (TNF) (Parkin & Cohen, 2001; Zhang *et al*, 2011). Mast cell activation via FcɛRI leads to mitochondrial fission and translocation of mitochondria to secretory granule exocytosis sites. Inhibition of DRP1 activity by Mdivi‐1 treatment decreased mitochondrial translocation and subsequent degranulation, indicating the importance of mitochondrial fission in the mast cell immune response (Fig 2) (Zhang *et al*, 2011).

### Mitochondrial dynamics in natural killer cells

Natural killer cells (NK cells) are innate lymphocytes that are crucial for anti‐tumour and anti‐viral immune responses. Numerous studies have implicated mitochondrial dynamics in the anti‐cancer function of NK cells. With regard to infection, NK cells have an important role in immune control of human immunodeficiency virus (HIV‐1) infection, and again mitochondrial dynamics may impact this response. NK cells isolated from HIV‐1/human cytomegalovirus (HCMV) seropositive donors exhibited small spherical mitochondrial shape, whereas those from HIV‐1/HCMV seronegative donors showed long and tubular mitochondrial morphology (Fig 2) (preprint: Cubero *et al*, 2019). The direct implications of mitochondrial dynamics of NK cell function in HIV infection are still unclear. However, it highlights an emerging new area in which changes in mitochondrial dynamics may underpin NK anti‐viral function and thus warrants further investigation.

### Mitochondrial dynamics in monocytes

Monocytes are phagocytes which participate in the innate immune response and have the potential to differentiate into macrophages and dendritic cells. The plasticity of monocytes allows for a tailored immune response depending on many factors such as Toll‐like receptor (TLR) stimulation, and mitochondria are crucial for monocyte adaptation to environmental cues (Merah‐Mourah *et al*, 2020). During TLR4 stimulation with lipopolysaccharide (LPS), CD14+ human monocytes become activated which involved reduced expression of MTFP1 and increased mitochondrial fusion. This was required for the metabolic adaptation of monocytes to LPS stimulation, necessary for pro‐inflammatory cytokine and chemokine expression (Duroux‐Richard *et al*, 2016). Moreover, mitochondrial elongation is also observed during murine monocyte to macrophage differentiation (Li *et al*, 2020). Monocytes are a heterogenous population of cells, further studies are needed to determine the exact role of mitochondrial dynamics in each of the different subsets.

### Mitochondrial dynamics in macrophages

Macrophages are long‐lived key innate immune cells and were first described by Metchnikoff well over 100 years ago (Metchnikoff, 1883). They are multifunctional phagocytes, which can detect, engulf and destroy pathogens and apoptotic cells. They are the main producers of cytokines and are involved in resolution of inflammation and tissue repair. Due to their range of functions throughout the inflammatory response to infection, it is unsurprising that their dysfunction contributes significantly to the pathogenesis of a range of inflammatory and degenerative diseases (Wynn *et al*, 2013). Activated macrophages are traditionally divided into two categories, M1‐like versus M2‐like. M1 macrophages are mainly involved in pro‐inflammatory responses and the spectrum of M2 macrophages are mainly involved in anti‐inflammatory, pro‐resolution responses, although these definitions are strongly oversimplified as macrophage phenotype is vastly more complex *in vivo* (Mosser & Edwards, 2008; Orecchioni *et al*, 2019). Bone marrow‐derived macrophages (BMDMs) are often stimulated with TLR4 ligand LPS as a model of M1 polarisation. In this scenario, time‐lapse microscopy showed that mitochondria switched to a fragmented morphology within 2 hours of LPS stimulation, and expression of fusion mitochondrial proteins MFN1, MFN2 and phosphorylation of DRP1 (Ser637) was suppressed. This increase in fragmentation also correlated with an increase in mitochondrial mtROS production (Gao *et al*, 2017). IL‐4 stimulation of BMDMs is known to promote M2 polarisation and which causes macrophage mitochondria to form elongated tubules occupying more cytoplasmic area in the cell (Fig 2) (Gao *et al*, 2017).

### Mitochondrial dynamics and macrophage function

Studies have shown that mitochondrial dynamics are also critical in macrophage function. For example, increased mitochondrial fusion and mitochondrial activity precedes increased macrophage phagocytic and bactericidal activity against *Salmonella typhimurium* infection (Fig 2) (Oliva‐Ramírez *et al*, 2014). Macrophages can prevent post‐apoptotic necrosis and dampen inflammation through clearance of apoptotic cells by phagocytosis, a process known as efferocytosis. Macrophage uptake of multiple apoptotic cells requires DRP1‐mediated mitochondrial fission, as silencing *Drp1* led to impaired efferocytosis in these macrophages. Inhibition of mitochondrial fission led to a striking increase of necrotic core area and the accumulation of apoptotic cells *in vitro* and in thymus tissue *in vivo*. Therefore, changes in mitochondrial dynamics underpin macrophage efferocytosis, a process which is critical for normal tissue homeostasis (Wang *et al*, 2017).

### Mitochondrial dynamics and the macrophage circadian clock

What has become increasing clear over the past decade is that macrophages are highly controlled by our molecular clock. This molecular clock imparts 24 rhythms, or circadian rhythms in biological function so as to align with the daily changes in our environment. The breadth of circadian control over both monocyte and macrophage function has been reviewed in detail here (Timmons *et al*, 2020). Circadian rhythms provide a selective advantage by anticipating organismal nutrient needs and guaranteeing optimal metabolic capacity and performance during active versus rest phases. We now observe that the macrophage immune response to both bacterial and viral infection is conditioned by time of day of exposure (Bellet *et al*, 2013; Sengupta *et al*, 2019). Remarkably, mouse macrophages alter their mitochondrial morphology over the course of the day, such that mitochondria are more fused during the rest phase and more fragmented during the active phase. This change in morphology also maps to circadian changes in metabolism such as ATP production and function such as phagocytosis (Collins *et al*, 2021). Further research is needed to fully understand the exact impact of these daily rhythms in macrophage mitochondria morphology and how this may impact the macrophage response to infection at discrete times of day. Further work is also required to understand the impact of circadian disruption, which is evident in shift workers, on this rhythmic cycling of mitochondrial dynamics in macrophages and also what additional immune cells also possess this intriguing feature.

### Mitochondrial dynamics in NLRP3 inflammasome activation

The inflammasome is a multiprotein intracellular complex and is one of the most important mechanisms for innate immune defence against microbial infection. The inflammasome detects pathogenic microorganisms and damage‐associated molecular patterns (DAMPs) such as mtROS, which activates the highly pro‐inflammatory cytokines interleukin‐1β (IL‐1β) and IL‐18. The role of mitochondria in regulating the activation of the NLR family pyrin domain‐containing 3 (NLRP3) inflammasome has garnered much interest in recent years and have been extensively reviewed (Liu *et al*, 2018). In macrophages, knockdown of *Drp1* led to aberrant mitochondrial elongation and increased NLRP3‐dependent caspase‐1 activation and IL‐1β secretion. NLRP3 inflammasome assembly is determined by NLRP3‐ASC interaction and apoptosis‐associated speck‐like (ASC) oligomerisation, both of which were much stronger in *Drp1*‐knockdown BMDMs. Treatment with the mitochondrial ETC uncoupler carbonyl cyanide m‐chlorophenylhydrazone (CCCP) is known to induce mitochondrial fission in cells. Interestingly, treatment of BMDMs with CCCP led to mitochondrial fission, but also attenuated assembly and activation of the NLRP3 inflammasome (Park *et al*, 2015). Mitochondrial morphology also plays a role in terms of NLRP3 sensing and response to viral infection. Upon infection with RNA viruses such as influenza virus, NLRP3 and MAVS associate with MFN2 in a mitochondrial membrane potential (ΔΨm)‐dependent manner. MFN2 associates with the NLRP3 inflammasome through its central 4,3 hydrophobic heptad repeat region 1 (HR1) to recruit adaptor protein ASC and procaspase‐1 and form the NLRP3 inflammasome (Ichinohe *et al*, 2013). Sensing of single‐stranded RNA viruses such as influenza virus has also been shown to trigger activation of the serine‐threonine kinases RIP1 and RIP3. This promoted activation of DRP1 and its translocation to mitochondria to drive mitochondrial fission, induction of mtROS, the formation of mitochondrial aggregates in the perinuclear space, and activation of the NLRP3 inflammasome (Wang *et al*, 2014). These results are in contrast to the aforementioned study which indicated mitochondrial elongation is necessary for NLRP3 inflammasome assembly (Park *et al*, 2015). As we will see later on, different pathogens modulate mitochondrial dynamics through various mechanisms. Nevertheless, these results demonstrate the importance of mitochondrial dynamics in NLRP3 inflammasome assembly and activation, a crucial function of macrophages during infection.

### Mitochondrial dynamics in dendritic cells

Dendritic cells (DCs) specialise in antigen recognition, uptake and processing, and subsequent antigen presentation to T cells. As such, DCs are the bridge between innate and adaptative immune responses. DCs are classified into different subsets depending on their origin, localisation and function, and they are strategically located throughout the body as the first line of pathogen sensing. In humans, there are two main subsets of DC: myeloid DCs (mDCs) participate in anti‐bacterial and anti‐fungal immune responses, while plasmacytoid (pDCs) are involved in anti‐viral responses (Collin & Bigley, 2018). Mitochondrial metabolism plays a crucial role in DC differentiation (Zaccagnino *et al*, 2012), as metabolism directs the activation state and subtypes of DCs (Pearce & Everts, 2015).

Specifically regarding mitochondrial dynamics, there are very few studies on the role of fission and fusion in DC function. Mitochondrial fusion is induced during GM‐CSF‐stimulated differentiation of bone marrow progenitor cells to immature dendritic cells, which is accompanied by the upregulation of mitochondrial fusion proteins such as OPA1 and MFN2 (Fig 2) (Ryu *et al*, 2014). TLR7 and TLR8 stimulation increased protein expression of DRP1 and induced mitochondrial fragmentation mDCs. Meanwhile, in pDCs, TLR7/8 stimulation increased MFN2 expression and mitochondrial content. These findings reveal that TLR7/8 stimulation differentially regulates mitochondrial dynamics in distinct human DC subsets, which contributes to their activation (Basit *et al*, 2018). Interestingly, infection of BMDCs with influenza A virus (IAV) was sensed by Nucleotide‐binding oligomerisation domain‐containing protein 2 (NOD2) and RIPK2 and promoted mitophagy, which in turn cleared damaged mitochondria to avoid overactivation of NLRP3 inflammasome.

Upon interaction with T cells, DCs mobilise their mitochondria to the immune synapse which is the contact site between the DC and T cell. This is accompanied by partial depolarisation of membrane potential, high levels of mtROS, and mitophagy, which could be a homeostatic mechanism to control the quality of mitochondria in this region (Gómez‐Cabañas *et al*, 2019). Further investigations are needed to fully elucidate how mitochondrial dynamics affects DC function and the impact this has on the critical role of DCs and their interaction with T cells in bridging the innate and the adaptive immune response.

### Mitochondrial dynamics in T cells

Adaptive immunity is characterised by the use of antigen‐specific receptors on T and B cells to drive targeted adaptive effector responses. Firstly, antigens are presented to and recognised by antigen‐specific T or B cell, leading to cell priming, activation and differentiation, which usually occurs within the specialised environment of lymphoid tissue. Secondly, the effector response takes place due to activated T cells leaving the lymphoid tissue and homing to the disease site, or due to the release of antibodies from activated B cells into blood and tissue (Parkin & Cohen, 2001).

During infection, naïve T cells (T_N_) challenged with an antigen rapidly proliferate into effector T cells (T_E_). The majority of T_E_ cells undergo cell death, while a few long‐lived memory T cells (T_M_) remain after the infection diminishes. T_M_ cells can be reactivated into rapidly expanding T_E_ cells if a similar infection occurs in order to quickly curtail the infection. Regulatory T cells (Tregs) suppress the proliferation and function of effector T cells. T‐cell subtypes (T_N_, T_E_, T_M_ and Treg) have distinct functions and metabolic profiles which are intrinsically linked to changes in mitochondrial morphology (Weinberg *et al*, 2015).

Formation of the immune synapse (IS) is essential for T‐cell activation. The IS formation induces redistribution of many signalling molecules and it has been shown that the redistribution of mitochondria to the IS is required for T‐cell activation and subsequent differentiation (Quintana *et al*, 2007). Following an APC‐T‐cell interaction, DRP1 is recruited to the mitochondria to mediate mitochondrial positioning at the IS. Upon *Drp1* knockdown in T cells, mitochondrial translocation towards the IS was reduced. Moreover, *Drp1* knockdown decreased membrane potential and ATP production. The phosphorylation of myosin is an ATP‐dependent process necessary for myosin activation and activated myosin is required for IS formation (Ilani *et al*, 2009). Therefore, *Drp1* and its control of mitochondrial morphology and ATP production through control of myosin directly impact IS organisation function. Activated T cells produce mtROS, and this process is also dependent on mitochondrial fission. mtROS are required in the activation of the transcription factor NF‐κB, which transcribes IL‐2. Inhibition of DRP1 by Mdivi‐1 reduced IL‐2 mRNA levels and T‐cell proliferation (Fig 2).

These results suggest that DRP1 and mitochondrial fission impact mitochondrial position and activity at the IS, T‐cell receptor (TCR) proximal signalling and IL‐2 production. This illustrates the fundamental role of mitochondrial dynamics for T‐cell biology and function (Baixauli *et al*, 2011).

T_E_ cells augment anabolic pathways of metabolism such as aerobic glycolysis, while T_M_ cells engage catabolic pathways, like fatty acid oxidation (FAO), and these metabolic differences are reflected in mitochondrial morphology. T_E_ cells have decreased expression of the fusion proteins MFN2 and OPA1 and high levels of active DRP1 (Ser616 phosphorylation), leading to punctate mitochondria. On the other hand, T_M_ cells have increased protein expression of MFN2 and OPA1 to maintain fused networks. In terms of T_M_ function, OPA1 appears crucial, as its deletion caused defects in T_M_ survival (Fig 2). Fusion in T_M_ cells configured ETC complex associations favouring OxPhos and FAO, while fission in T_E_ cells led to cristae expansion, reduced ETC efficiency which promoted aerobic glycolysis. Therefore, mitochondrial dynamics in T cells shapes the metabolic and cellular fate of T cells (Buck *et al*, 2016; Rambold & Pearce, 2018).

### Mitochondrial dynamics in B cells

B lymphocytes mature in the bone marrow and move through the lymphatic system to circulate throughout the body. Their main role is to generate antigen‐specific antibodies. B‐cell activation requires two main signals: firstly, the recognition of the antigen by the B‐cell antigen receptor (BCR), followed by a second signal provided by helper T cells (Th), which express the co‐stimulatory molecules CD40 and cytokines, such as IL‐4 and IL‐21. Collectively, this provides the necessary microenvironment for the activation and subsequent proliferation of B cells and antibody production (Crotty, 2015).

Mitochondrial remodelling occurs during B‐cell activation. A few studies have shown that mitochondrial mass and volume increased in B cells stimulated with CpG alone or CpG plus anti‐IgM in comparison with those B cells stimulated with anti‐IgM alone. Activated B cells increase glucose uptake, TCA cycle and OxPhos and have fragmented mitochondria (Fig 2), while naïve B cells maintain a predominance of elongated mitochondria (Caro‐Maldonado *et al*, 2014; Waters *et al*, 2018). These findings are similar to those conducted in T cells; however, it was found that naïve B cells have significantly fewer mitochondria in comparison to naïve T cells (Ron‐Harel *et al*, 2016). It is typically reported that elongated mitochondria are associated with increased OxPhos, while fragmented mitochondria have reduced OxPhos capacity (Galloway *et al*, 2012). Perhaps this morphological strategy is employed by B cells to support rapid, B‐cell proliferation and expansion to initiate a humoral immune response (Waters *et al*, 2018). These studies suggest how B‐cell metabolism adapts with stimulation and reveals unexpected details of mitochondrial dynamics at the start of a humoral immune response.

Although there is evidence of how mitochondrial dynamics are involved in the activation and differentiation of lymphocytes, future studies are needed to fully delineate why and how mitochondrial dynamics change in T and B cells as they go through stages of differentiation and activation and also the consequences of this on their cellular function.

## Mitochondrial dynamics in host immunity against infection

Mitochondria play essential roles in anti‐viral and anti‐bacterial signalling in cells, including the RLR and TLR signalling pathways to sense the presence of pathogens. Therefore, it is of no surprise that many pathogens have evolved strategies to manipulate fission and fusion to aid in their own survival (Tiku *et al*, 2020). Viruses and bacteria infect both “traditional” immune cells and “non‐traditional” host immune cells such as epithelial and endothelial cells to circumvent clearance by immune cells.

**Figure 3:**
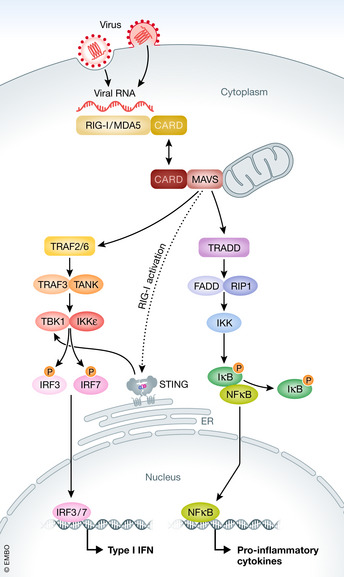
Viral detection and signalling pathways via RLR signalling RIG‐like receptors (RLR) detect viral RNA and activate downstream signalling pathways to initiate the immune response. RIG‐1 and MDA‐5 recognise and bind to viral mRNA which leads to activation of MAVS on the mitochondrial membrane. MAVS dimerises and associates with adaptor proteins such as TRAF2/6 and TRADD. This leads to the recruitment of TRAF3 and TANK, which activates TBK1 and IKK epsilon. IRF3 and IRF7 are phosphorylated by TBK1 and IKK epsilon, leading to homodimerisation and translocation of IRF3 and IRF7 to the nucleus where they promote the expression of type I IFNs. The association of TRADD with MAVS leads to the recruitment of FADD and RIP1 which activates IKK. IKK phosphorylates IkB which becomes ubiquinated and targeted for degradation, allowing NF‐kB to translocate to the nucleus where it promotes the expression of pro‐inflammatory genes. Viral sensing via RIG‐1 can also lead to the association of MAVS with STING, located at the ER, which has been shown to be important for IRF3 type I IFN production.

Viruses are obligate intracellular pathogens which hijack host cell machinery in order to survive and replicate. Viruses are reliant on host cells’ metabolism for energy requirements, and their modulation of molecular machinery and mitochondrial dynamics leads to alterations in organelle function. Mitochondria participate in host anti‐viral immunity through MAVS activation and mitochondrial dynamics. Viral pathogen‐associated molecular patterns (PAMPs) are recognised by intracellular pattern recognition receptors (PRRs) such as RIG‐I, MDA‐5 and LGP2, which sense viral RNA in the cytoplasm and activate MAVS through binding via CARD domains. MAVS is located in the OMM and once activated it associates with adaptor proteins such as TRAF2, TRAF6 and TRADD which recruits TRAF3 and TANK. This recruitment triggers the activation of TBK1 and IKK epsilon which promotes the phosphorylation and subsequent dimerisation of the transcription factors IRF3 and IRF7, necessary for the mRNA expression of type I IFNs. Concurrently, the MAVS/TRADD complex recruits RIP1 and FADD, leading to IKK activation. IKK phosphorylates the NF‐kB inhibitor IkBα, releasing NF‐kB which then drives pro‐inflammatory cytokine mRNA expression. MAVS is also known to interact with Stimulator of interferon genes (STING) and TOM70 in the mitochondria, both of which are required for IRF3 production of type I IFNs upon viral sensing by RIG‐I (Loo & Gale, 2011) (Fig 3).

Studies have shown that mitochondrial dynamics play an important role in the viral immune response, with elongation and fragmentation of mitochondria affecting RIG‐I‐like receptor (RLR) signalling. In epithelial cells, RLR signalling leads to mitochondrial elongation through interaction of MAVS with the mitochondrial fusion protein MFN1. Upon viral signalling, the higher isoform of MAVS becomes degraded, leading to the release of MFN1 which promotes mitochondrial fusion. Fusion allows for increased interaction between MAVS and STING, which enhances type I IFN expression. Cells with a mutated version of *Drp1* had elongated mitochondria and were also found to have enhanced activation of the IFN‐β promoter (Castanier *et al*, 2010). Similarly, another study found that MFN1 positively regulates anti‐viral responses, necessary for MAVS activation and subsequent viral signalling in mouse embryonic fibroblasts (MEFs) (Onoguchi *et al*, 2010). Conversely, loss of MFN2 was found to enhance anti‐viral signalling mediated by MAVS. Moreover, the same group found that MEFs lacking MFN1 and MFN2 had defective anti‐viral immune responses and a loss of mitochondrial membrane potential, with decreased MAVS signalling and subsequently impaired expression of IFN‐β and IL‐6 (Yasukawa *et al*, 2009).

These studies highlight the importance of mitochondrial dynamics in the viral immune response, by fine‐tuning RLR signalling via MAVS and subsequently impacting interferon synthesis and viral clearance. Clearly, mitochondrial dynamics is important in executing viral signalling pathways and inflammatory responses during infection. However, viruses have been able to take hijack some of these mechanisms in order to propagate their own survival. As we will see in the subsequent section, this involves increased mitochondrial fusion or fission depending on the virus and host cell type they infect. Below we outline several examples of viruses which specifically manipulate mitochondrial remodelling in host cells in order to enhance their own replication.

## SARS

**Figure 4:**
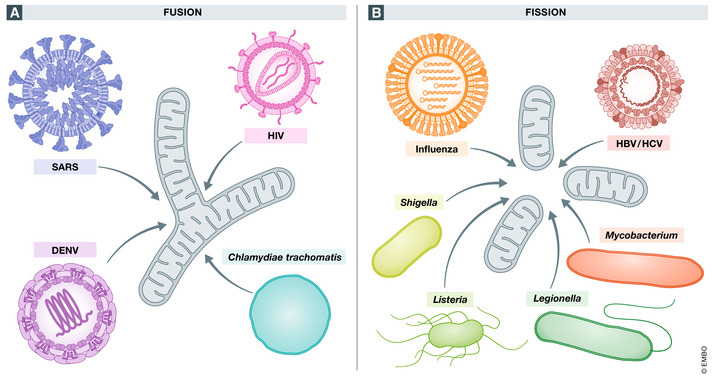
Mitochondrial dynamics in host immunity against infection (A) Mitochondria play essential roles in anti‐viral and anti‐bacterial signalling in cells. Viruses such as SARS, DENV and HIV enhance mitochondrial fusion to promote virus survival and inhibit the interferon response, and the intracellular bacteria *Chlamydiae trachomatis* also induces mitochondrial fusion to promote bacterial survival. (B) Other viruses such as influenza, hepatitis B and hepatitis C induce mitochondrial dysfunction via enhanced mitochondrial fragmentation leading to viral persistence in cells. Bacterial infection from *Listeria monocytogenes*, *Legionella pneumophilia*, *Mycobacterium tuberculosis* and *Shigella flexneri* have evolved mechanisms of circumventing clearance by manipulating host cell mitochondrial dynamics to maintain a favourable environment for pathogen survival.

Severe acute respiratory syndrome (SARS) is a viral respiratory disease caused by (+)ssRNA SARS‐associated coronaviruses, including SARS‐CoV‐1 and more recently SARS‐CoV‐2. These coronaviruses infect epithelial cells in the lungs by binding the ACE2 receptor. SARS‐CoV‐1 disturbs type I IFN pathways in order to circumvent clearance. The genome of SARS‐CoV‐1 encodes an accessory protein ORF‐9b, which has been shown to have a functional role in altering host cell mitochondrial dynamics and manipulating interferon signalling pathway components, disrupting the inflammatory response to the virus. Expression of ORF‐9b in HEK293T cells and THP‐1 cells led to a reduction in DRP1 levels and increased mitochondrial elongation driven by MFN2 (Fig 4A) (Shi *et al*, 2014). As mentioned previously, MFN2 inhibits MAVS which increases IFN production (Yasukawa *et al*, 2009), indicating that SARS‐CoV‐1 employs ORF‐9b to induce fusion and decrease the IFN response. Moreover, ORF‐9b was found to bind to MAVS, leading to MAVS degradation, disrupting anti‐viral signalling and subsequently IFN production (Shi *et al*, 2014). Thus, SARS‐CoV‐1 modulates mitochondrial dynamics in host cells in order to reduce the type I IFN response and propagate its survival.

SARS‐CoV‐2 has also been implicated in manipulating mitochondrial dynamics in host cells. SARS‐CoV‐2 also expresses ORF‐9b, which was reported to interact with TOM70, a subunit which recruits MAVS pathway components to the OMM (Gordon *et al*, 2020). This may have the same effect on host cell mitochondrial dynamics as ORF‐9b expression in SARS‐CoV‐1. Moreover, SARS‐CoV‐2 also expresses ORF‐9c which reportedly interacts with components of the ETC, suggesting a possible role of ORF‐9c in manipulating host cell OxPhos (Gordon *et al*, 2020).

### Dengue virus

Dengue virus (DENV) is a mosquito‐borne (+)ssRNA flavivirus and the causative agent of dengue fever. DENV targets myeloid‐derived cells such as monocytes, macrophages and dendritic cells, as well as endothelial cells and hepatocytes.

DENV possesses the protease NS2B3 which can cleave STING, an adaptor protein required for IFN signalling. By cleaving STING, DENV circumvents the innate immune response. One study found that on top of targeting STING, DENV protease NS2B3 also targets MFN1 and MFN2 for degradation, which was found to impair mitochondrial fusion and IFN signalling in epithelial cell lines (Fig 4A) (Yu *et al*, 2015).

Conversely, infection of hepatoma cells with DENV led to mitochondrial elongation by reducing DRP1Ser616 phosphorylation. By inducing fusion, DENV infection led to the formation of convoluted membranes from the ER, leading to disrupted contact between the ER and mitochondria which ultimately reduced MAVS signalling, inhibiting the anti‐viral response (Chatel‐Chaix *et al*, 2016). Similarly, DENV infection of hepatoma cells and monocyte‐derived DCs induced mitochondrial elongation via DRP1 inhibition, with increased mitochondrial respiration and ATP production. Conversely, when mitochondrial fragmentation was reversibly induced with CCCP in epithelial cells, viral replication was inhibited. Removal of CCCP resulted in increased elongation and viral titres to levels in untreated DENV‐infected epithelial cells (Barbier *et al*, 2017).

DENV manipulates mitochondrial dynamics of host cells in order to promote its survival; however, the mechanism employed and outcome appears to be cell type‐dependent. Therefore, more extensive investigations employing all the different cell types which DENV infects is needed to fully appreciate how DENV influences mitochondrial remodelling.

### Influenza A infection

Influenza A viruses (IAV) are (‐)ssRNA viruses and are responsible for global pandemics including the 1918 flu pandemic and the 2009 swine flu pandemic, as well influenza epidemics which occur each year. IAV employs numerous mechanisms to promote its survival in epithelial cells, including manipulation of mitochondrial dynamics. In infected cells, the viral protein PB1‐F2 binds to MAVS to inhibit RLR signalling and thus reduce anti‐viral immunity (Varga *et al*, 2012). Furthermore, PB1‐F2 translocates to the mitochondria leading to increased mitochondrial fission and loss of membrane potential (Fig 4B) (Zamarin *et al*, 2005; Yoshizumi *et al*, 2014). Knockdown of *Drp1* inhibited mitochondrial fission induced by PB1‐F2. The *Drp1*‐dependent changes in host cell mitochondrial morphology correlated with impaired RIG‐I signalling and inhibition of the NLRP3 inflammasome (Yoshizumi *et al*, 2014).

In contrast to the above findings, a recent study found that infection of epithelial cells with IAV unexpectedly induced mitochondrial elongation. Elongation was observed with infection using H1N1, H3N2 and influenza B strain. Transfection of cells with influenza RNA was sufficient to induce mitochondrial elongation, decrease DRP1 presence at the mitochondria and propagate the IFN response. Treatment of IAV‐infected epithelial cells with the fission‐inducing molecule Mito‐C reduced viral replication and reversed IAV‐induced elongation in infected cells (Pila‐Castellanos *et al*, 2021). The differences between the effects of IAV infection in epithelial cells between the above studies may be due to experimental conditions and varying degrees of cellular stress which can impact mitochondrial morphology. Therefore, it appears that IAV targets mitochondrial‐dependent mechanisms and morphology to subvert host cell anti‐viral responses and propagate its survival in host cells.

### Hepatitis B and C viruses

Modulation of mitochondrial dynamics has been observed both for hepatitis B (HBV) and hepatitis C (HCV) viruses. Both HBV and HCV infect hepatocytes, leading to chronic liver damage and eventually hepatocellular carcinoma (HCC). Importantly, infection of host cells with HBV and HCV leads to dysregulation of mtROS generation, Ca^2+^ signalling and mitochondrial dysfunction. Infection of hepatocytes with HBV or HCV induced mitochondrial fragmentation via phosphorylation of DRP1 (Ser616), leading to DRP1 translocation to the mitochondria and mitophagy, which in turn inhibited mitochondrial induced apoptosis (Fig 4B). Moreover, the HCV protein NS5A has been reported to colocalise with and cleave MAVS in the mitochondria of infected hepatocytes, leading to disruption of IFN signalling in cells (Refolo *et al*, 2019), while other core HCV proteins can induce phosphorylation of DRP1 (Ser616) (Kim *et al*, 2014). Inhibiting DRP1 activity, thus promoting fusion, led to increased apoptosis and increased IFN synthesis in HCV‐infected cells, suggesting that mitochondrial fission promoted viral persistence in cells. These viruses can manipulate its host cell through the mitochondria to prevent cellular apoptosis. This intelligent strategy of preventing cell death in infected cells leads to viral persistence of HBV and HCV (Kim *et al*, 2013, 2014).

Overall these studies demonstrate that both HBV and HCV employ similar mechanisms to modulate host cell mitochondrial dynamics in order to subvert anti‐viral responses.

Therefore, viruses manipulate host cell mitochondrial dynamics to impact host cell mitophagy, apoptosis, as well as anti‐viral signalling. These physiological changes caused by disrupted mitochondrial dynamics severely impact the immune systems’ ability to clear the virus, leading to persistent infection. Further studies are necessary to fully tease apart exactly how certain viruses manipulate host cell mitochondrial dynamics to circumvent the immune response. A greater understanding in this area may lead to the development of much needed new anti‐viral drugs.

### Mitochondrial dynamics in host immunity against bacterial infection

Given the importance of mitochondria in the anti‐bacterial response, it is of no surprise that several bacteria have evolved mechanisms of circumventing clearance by manipulating host cell mitochondrial dynamics to maintain a favourable environment for pathogen survival. Most bacteria which target mitochondrial remodelling tend to be intracellular (although there are some exceptions) and most of the strategies employed by bacteria lead to mitochondrial fission as we will outline in this section. There have been numerous studies to date outlining the role of mitochondria in innate immune signalling, and macrophages are the main players in the anti‐bacterial response. TLR signalling is a pivotal component of the innate immune response, sensing PAMPs and DAMPs during infection ultimately leading to the production of pro‐inflammatory mediators required to clear pathogens from the body. Mitochondria play a role in augmenting the inflammatory response upon TLR stimulation (West *et al*, 2011). For example, upon TLR4 stimulation by LPS, macrophages undergo a metabolic switch from OxPhos to glycolysis. This metabolic change to glycolysis is accompanied with an increase in fragmented mitochondria and increased mtROS production, which is crucial for bacterial clearance. This increase in mtROS production occurs through TLR recruitment of mitochondria to phagosomes, where TRAF6 translocates to the mitochondria. Macrophages lacking TRAF6 have reduced bactericidal activity, demonstrating the importance of translocation of TRAF6 to the mitochondria upon TLR stimulation. Mitochondria also participate in TLR1‐ and TLR2‐mediated signalling (gram‐negative bacteria and mycoplasma sensing) in a similar manner to TLR4 (West *et al*, 2011).

Below we outline how several bacteria manipulate host cell mitochondrial dynamics in order to create an appropriate intracellular niche for bacterial survival and replication.

### Listeria monocytogenes

Most early work investigating bacterial manipulation of mitochondrial dynamics in host cells was carried out using *Listeria monocytogenes*. *L*. *monocytogenes* is a gram‐positive, facultative anaerobic intracellular bacterium well known for causing the disease listeriosis. *L*. *monocytogenes* invades epithelial cells, while macrophages also internalise *L*. *monocytogenes* via phagocytosis. Once internalised, the bacterium must escape the phagosome in order to survive in the cytosol. It does so by producing a pore‐forming toxin known as listeriolysin O (LLO). Upon lysis of the phagosome, *L*. *monocytogenes* escapes into the cytosol where it hijacks the host cell’s actin filaments via the bacterial protein ActA. This allows the bacterium the ability to move within the cell and subsequently spread to other cells.

*L. monocytogenes* can dramatically alter the mitochondrial dynamics of its host cell, *L*. *monocytogenes* infection induces mitochondrial fragmentation in the host cell mitochondria (Fig 4B) (Stavru *et al,* 2011). Mitochondrial fragmentation was found to be dependent on Ca2+ influx into the mitochondria which led to a decrease in mitochondrial respiration, membrane potential and intracellular ATP levels. These changes were caused by the pore‐forming actions of LLO and were necessary for efficient *L*. *monocytogenes* infection. Interestingly, fragmentation of the host cell mitochondria only occurred in early stages of infection, with no observed alterations of mitochondrial architecture at late stages of infection. Inducing mitochondrial fragmentation in cells prior to infection strongly impaired *L*. *monocytogenes* infection, while more infection was observed in host cells with hyperfused mitochondria. The conclusion from this study was that mitochondria which are more fused allows for *L*. *monocytogenes* invasion, and upon entry into the cell *L*. *monocytogenes* induces fission to impair the host cell’s ability to clear the pathogen by reducing intracellular ATP levels (Stavru *et al*, 2011).

### Legionella pneumophilia

*Legionella pneumophilia* is a gram‐negative aerobic bacterium and the causative agent of a severe form of acute pneumonia known as Legionnaires’ disease. First described over 40 years ago, this intracellular bacterium infects alveolar macrophages and epithelial cells and replicates in vacuoles following internalisation.

During infection of human monocyte‐derived macrophages (hMDMs), *L*. *pneumophilia* uses a type IV secretion system (T4SS) in order to manipulate host cell mitochondrial dynamics to promote an environment suitable for its replication. This process involves the T4SS effector MitF which induced DRP1‐dependent mitochondrial fragmentation (Fig 4B) and leads to a Warburg‐like phenotype in the host cell, with decreased mitochondrial respiration and increased glycolysis. MitF is also a known activator of host cell Ran GTPase and has been shown to manipulate the host cell’s cytoskeleton and cell migration upon infection, and it is thought to execute mitochondrial fragmentation through interaction with this GTPase. Silencing DRP1 was found to reduce bacteria‐induced fragmentation, as well as reducing replication of *L*. *pneumophilia* (Escoll *et al*, 2017). Therefore, like *L*. *Monocytogenes*, *L*. *pneumophilia* induces fragmentation in host cell mitochondria to promote its survival in the cell.

### Mycobacterium tuberculosis

*Mycobacterium tuberculosis* is a facultative intracellular bacterium which infects monocytes and alveolar macrophages in the lungs and the causative agent of tuberculosis. Once phagocytosed, the bacterium prevents the acidification and fusion of the phagosome with the lysosome, allowing its survival and replication until it initiates necrosis in order to spread to other cells.

Aguilar‐Lopez *et al* found that two *M. tuberculosis* virulence factors, LprG and PE_PGRS33, had opposing effects on hMDM mitochondrial morphology. LprG is known to be immunosuppressive as administration with *M. tuberculosis* in mice leads to exacerbated infection and decreased survival, while PE_PGRS33 plays a role in the entry of *M. tuberculosis* into macrophages via TLR2. Stimulation of hMDMs with LprG led to mitochondrial fragmentation (Fig 4B), reduced oxygen consumption and a lower respiratory capacity compared to control cells and affected mitochondrial calcium uptake. Conversely, PE_PGRS33 induced mitochondrial fusion with no differences in respiratory capacity or calcium uptake. The authors suggest that the bacterial virulence factors may act on mitochondria at different stages of infection. Macrophage infection with *M. tuberculosis* leads to Warburg metabolism, with increased glucose uptake and glycolytic enzyme activity, which is necessary for pathogen clearance (Aguilar‐López *et al*, 2019). Perhaps mitochondrial fragmentation is first induced by intracellular bacterium in order to provide a suitable environment for effective infection and survival by impairing host cell metabolism, while in later stages fragmentation is reduced in order not to damage the infected host cell enabling persistent infection.

### Other bacteria

Many other bacteria appear to manipulate mitochondrial dynamics to promote their survival. *Chlamydiae trachomatis* is an obligate intracellular pathogen which infects epithelial cells and causes trachoma. Mitochondria are a key target of *C. trachomatis* due to the role of mitochondria in cell bioenergetics and apoptosis. Cells infected with *C. trachomatis* display aberrant metabolism, with increased NADPH consumption and lipid biosynthesis. Upon infection of epithelial cells, *C. trachomatis* elevated intracellular cAMP levels which was followed by phosphorylation of DRP1 (Ser637) leading to mitochondrial elongation (Fig 4A), increased ATP production. These mitochondrial changes led to increased *C. trachomatis* growth (Liang *et al*, 2018; Kurihara *et al*, 2019). Mitochondrial elongation occurred during early infection, with late stage infection characterised by fragmented mitochondria (Kurihara *et al*, 2019; Rother *et al*, 2019). Other studies have found mitochondrial elongation occurs during later stages of infection (Chowdhury *et al*, 2017) which may be due to differences in host cells employed as well as differences in culture medium and techniques.

*Shigella flexneri* is a gram‐negative intracellular bacteria and the causative agent of bacillary dysentery known as shigellosis. *S*. *flexneri* infects myeloid immune cells as well as intestinal epithelial cells. In HeLa cells, *S*. *flexneri* induces mitochondrial fragmentation through DRP1 (Fig 4B). Silencing of *Drp1* was found to reduce the ability of *S*. *flexneri* to spread from cell‐to‐cell and reduce the size of *S*. *flexneri* plaques (Lum & Morona, 2014).

Therefore, in summarising the literature, it is becoming clear that altering mitochondrial morphology underpins the capacity of a cell to mount an anti‐viral or anti‐bacterial response. As such many pathogens have developed their own mechanisms of manipulating host cell mitochondria and morphology as a counter measure to subvert clearance and allow their survival. Simultaneously, the host cell is continually alter its morphology for anti‐pathogen signalling at the different stages of infection. The winner of this battle determines the outcome of infection, whether a successful immune response is executed or whether the pathogen successfully circumvents clearance. However, it should be noted that the majority of studies discussed use *in vitro* model systems. Far less is known about pathogen infection on host cell mitochondrial dynamics in an *in vivo* setting and this requires significant investigation. Therefore, this area of research holds promise to offer a new range of therapeutics centred around remodelling host cell mitochondria to enhance pathogen clearance.

### Controlling mitochondrial dynamics as a therapeutic approach

Given that mitochondrial dynamic homeostasis is essential for normal cellular functions, mitochondrial remodelling has the potential to be a valuable therapeutic target in treating recurrent and persistent bacterial and viral infections (Table 1).

**Table 1:** Possible therapeutic approaches.

Compound	Promotes	Action	Functional effect	Reference
Mito‐C	Fission	Recruitment of DRP1	Anti‐viral effect Inhibits dengue virus replication Inhibits IAV replication	Molino *et al* (2020); Pila‐Castellanos *et al* (2021)
Mdivi‐1	Fusion	DRP1 Inhibitor	Alleviates kidney damage in a model of cell sepsis‐induced acute kidney injury Prevents degranulation of mast cells in allergies and atopic dermatitis	Liu *et al* (2020); Zhang *et al* (2011)
P110	Fusion	DRP1 Inhibitor	Protective against in vitro and in vivo models of septic cardiomyopathy	Haileselassie *et al* (2019)
MitoQ	Fusion	Antioxidant	Protective against 6‐OHDA‐induced mitochondrial fission	Solesio *et al* (2013)
Melatonin	Fusion	Antioxidant	Potential adjuvant in COVID‐19 vaccines to enhance mitochondrial quality	Zhang *et al* (2020)
BGP‐15	Fusion	Hydroxylamine derivative	Protects lung structure in a pulmonary arterial hypertension in vivo model	Szabo *et al* (2018)
6‐phenylhexanamide derivative mitofusin activators	Fusion	MFN activator	Induces fusion in neurons Protective against CMT2A	Dang *et al* (2020)

Recently, it has been shown that the pro‐fission compound Mito‐C has anti‐viral effects on cells through altering mitochondrial morphology to increase mitochondrial‐ER contact sites (Molino *et al*, 2020). Mito‐C was found to promote recruitment of DRP1 to mitochondria‐ER sites to induce mitochondrial fragmentation which inhibited dengue virus replication in host cells. Another study found that IAV infection induced mitochondrial elongation in epithelial cells which reduced mitochondrial‐ER contact sites and also anti‐viral signalling. Again, treatment of IAV‐infected cells with Mito‐C induced mitochondrial fragmentation and inhibited IAV replication. The increased mitochondria‐ER contact sites induced by Mito‐C treatment correlated with enhanced IFN activation through the RIG‐I signalling pathway (Pila‐Castellanos *et al*, 2021). Thus, Mito‐C may have broad anti‐viral properties against viruses which induce mitochondrial elongation and are sensed via RIG‐I. For example, SARS‐CoV‐1 and SARS‐CoV‐2 both express ORF‐9b which induces mitochondrial elongation upon epithelial cell infection. Mito‐C may therefore prove effective in inhibiting SARS‐CoV‐1/2 replication via modulating mitochondrial dynamics in host cells.

Mdivi‐1 is a well‐documented inhibitor of DRP1 and modulator of mitochondrial dynamics in cells. In numerous infection models, inhibition of DRP1 activity with Mdivi‐1 may be a viable therapeutic option. In a model of cell sepsis‐induced acute kidney injury (S‐AKI), Mdivi‐1 treatment inhibited mitochondrial fragmentation which prevented the release of mitochondrial contents and decreased NLRP3 activation in renal tubular epithelial cells. Mdivi‐1 treatment also alleviated kidney damage in LPS‐induced S‐AKI mice (Liu *et al*, 2020). The NLRP3 inflammasome is now a target of significant interest in the treatment of inflammatory diseases (Coll *et al*, 2015), and NLRP3 inflammasome activation serves as a critical step in the inflammatory response against pathogens. Targeting NLRP3 inflammasome activity in different cell types through modulation of mitochondrial dynamics may therefore represent another therapeutic option. Mdivi‐1 may also be useful in the treatment of allergies and atopic dermatitis, as inhibition of mitochondrial fission in mast cells prevented degranulation and TNF secretion from cells (Zhang *et al*, 2011). It should be noted, however, that in certain cell types, Mdivi‐1 inhibited mitochondrial complex I, impairing mitochondrial respiration with no mitochondrial elongation or reduction in DRP1 GTPase activity reported (Bordt *et al*, 2017). Therefore, the effects of Mdivi‐1 treatment may depend on cell type, which should be considered when focusing on mitochondrial dynamic‐based therapies. Inhibition of DRP1 and mitochondrial fission in cardiomyocytes with P110 has been shown to be protective against *in vitro* and *in vivo* models of septic cardiomyopathy (Haileselassie *et al*, 2019). Therefore, pharmacological modulation of mitochondrial dynamics is a viable therapeutic option. The protective effects of fission or fusion depend on the type of infection, target cell type or inflammatory condition.

Melatonin is known to prevent mitochondrial damage by promoting mitochondrial fusion, which limits mitochondrial fission induced oxidative stress and enhancing mitochondrial quality. Melatonin limits oxidative stress by enhancing anti‐oxidative enzymes as well as scavenging harmful mtROS and stimulates ATP synthesis in cells. A recent study suggested melatonin may be a potential adjuvant for treatment of COVID‐19 by enhancing mitochondrial quality and dampen hyper‐inflammatory responses to infection by the virus (Zhang *et al*, 2020).

MitoQ is an orally active antioxidant which targets mitochondrial dysfunction in an in vitro model of Parkinson's disease induced by 6‐hydroxydopamine (6‐OHDA) administration. Pre‐treatment of neuronal cells with MitoQ inhibited mitochondrial translocation of DRP1 and abrogated 6‐OHDA‐induced mitochondrial fission (Solesio *et al*, 2013). This study provides a compelling insight into MitoQ as a therapy for diseases involving mitochondrial fragmentation and oxidative stress. It would be interesting to investigate the potential of MitoQ in limiting the spread of pathogens which induce a fragmented phenotype in host cells for survival. BGP‐15 is a hydroxylamine derivative and has been shown to have extensivecytoprotective effects including reducing oxidative stress and increasing mitochondrial biogenesis. Recently, it has been demonstrated that BGP‐15 promotes mitochondrial fusion by activating optic atrophy 1 (OPA1), preventing mtROS induced fragmentation in multiple cell lines and in an *in vivo* model of pulmonary arterial hypertension (PAH). Mitochondrial fragmentation is an important factor in the development and progression of multiple diseases including PAH. Treatment of BGP‐15 in a mouse model of PAH induced mitochondrial fusion with a consequent reduction of inflammatory cell infiltration into the lungs (Szabo *et al*, 2018). This study provides therapeutic evidence of BGP‐15, which may also exert protective effects through modulation of mitochondrial morphology against viral and bacterial infections in host cells.

Finally, it has been shown that pharmacologically targeting MFN1 and MFN2 using 6‐phenylhexanamide derivative mitofusin activators induces fusion in neurons. Use of these activators was shown to reverse mitochondrial dysfunction in nerve neurons in a murine model of Charcot‐Marie‐Tooth disease type 2A (CMT2A), a genetic mutation which impairs MFN2 function and thus mitochondrial fusion (Dang *et al*, 2020). The promising effects of this compound in treating genetic diseases such as CMT2A suggests that activating MFN1/MFN2 may be a viable therapeutic option for reducing infection in instances where a pathogen induces mitochondrial fragmentation for survival.

## Conclusions and future directions

It has become increasingly clear that mitochondria perform in numerous ways in addition to their classical label as “the powerhouse of the cell”. The last two decades of research has clearly shown the importance of mitochondrial dynamics in immune cell function and how this organelle is central to anti‐bacterial and anti‐viral signalling pathways. Due to the importance and efficiency of mitochondria in pathogen clearance, numerous bacteria and viruses have evolved mechanisms to hijack mitochondrial dynamics for their own survival. In doing so, these microorganisms are manipulating mitochondrial dynamic machinery in an attempt to create a suitable environment in which to replicate and to hide intracellularly from patrolling immune cells. Understanding the biology underlying this battle between microorganisms and this organelle, which may itself be a descendent of bacteria, may provide a new class of anti‐bacterial, anti‐viral and immunomodulatory targets (see Box 1).

Box 1 **In need of answers**

- Why do distinct pathogens differentially manipulate mitochondrial dynamics in varied host cells?
- Can mitochondrial dynamics in host cells be therapeutically targeted to make host cells less viable for pathogenic infection?
- Can the mitochondrial dynamic machinery in immune cells be targeted to enhance innate and adaptive responses to limit infection?
- Can we elucidate the missing mechanisms of mitochondrial fission and fusion through studying pathogen manipulation of mitochondrial dynamics?

## Conflict of interest

The authors declare that they have no conflict of interest.

## Supporting information



Source Data for Figure 1
