# Supplementary material for: Alterations in mitochondrial morphology as a key driver of immunity and host defence
Source: EMBO Rep. 2021 Aug 2;22(9):e53086. doi: 10.15252/embr.202153086 (PMC8447557; doi:10.15252/embr.202153086)
Supplement: Supplementary file 1 — Source Data for Figure 1 [file EMBR-22-e53086-s001.pdf]

Figure 1

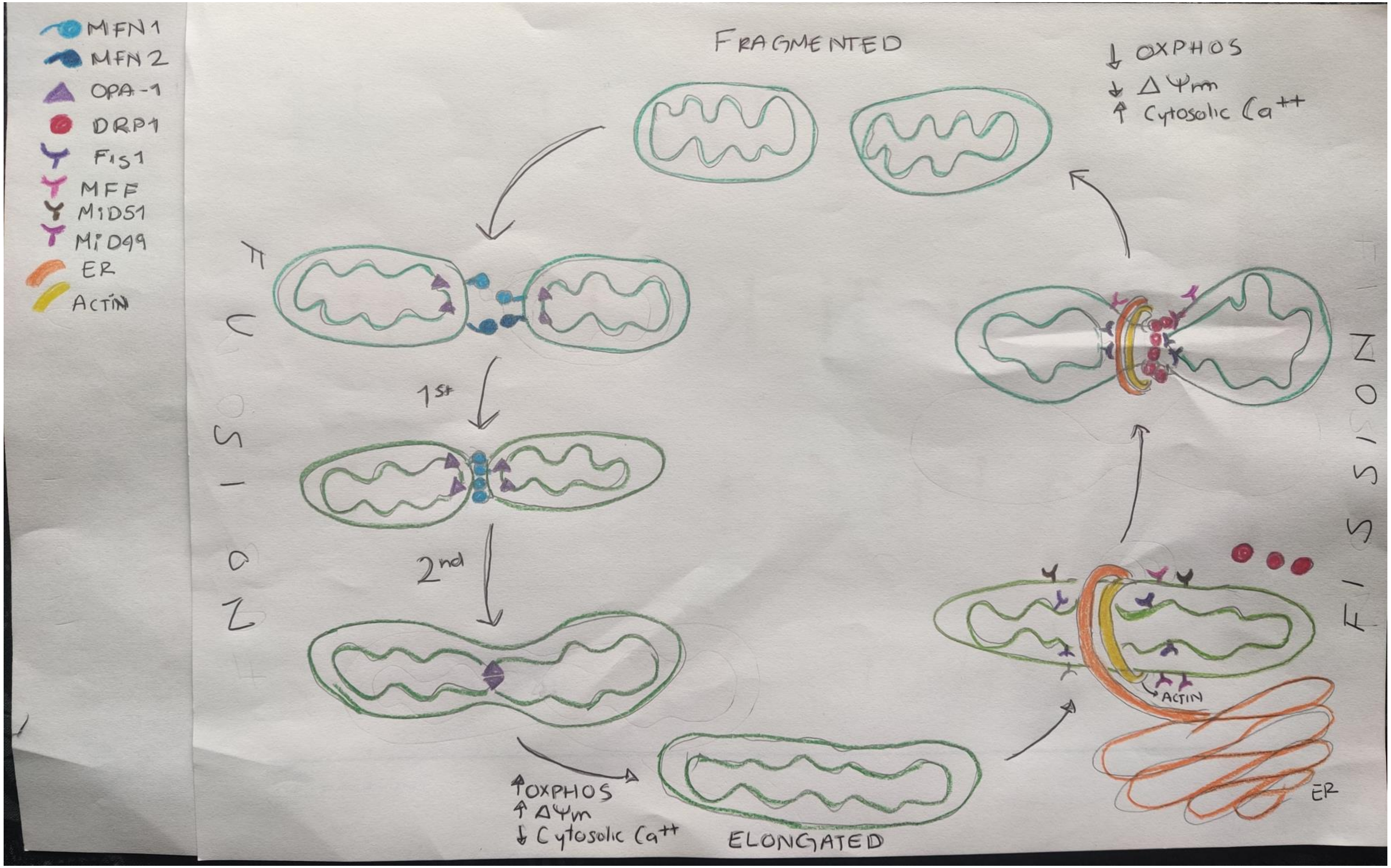
MFN1
MFN2
OPA-1
DRP1
Fis1
MFF
MiD51
MiD49
ER
ACTIN
FRAGMENTED
↓ OXPHOS
↓ ΔΨm
↑ Cytosolic Ca++
FUSION
1st
2nd
FISSION
ACTIN
ER
↑OXPHOS
↑ΔΨm
↓Cytosolic Ca++
ELONGATED

Figure 2

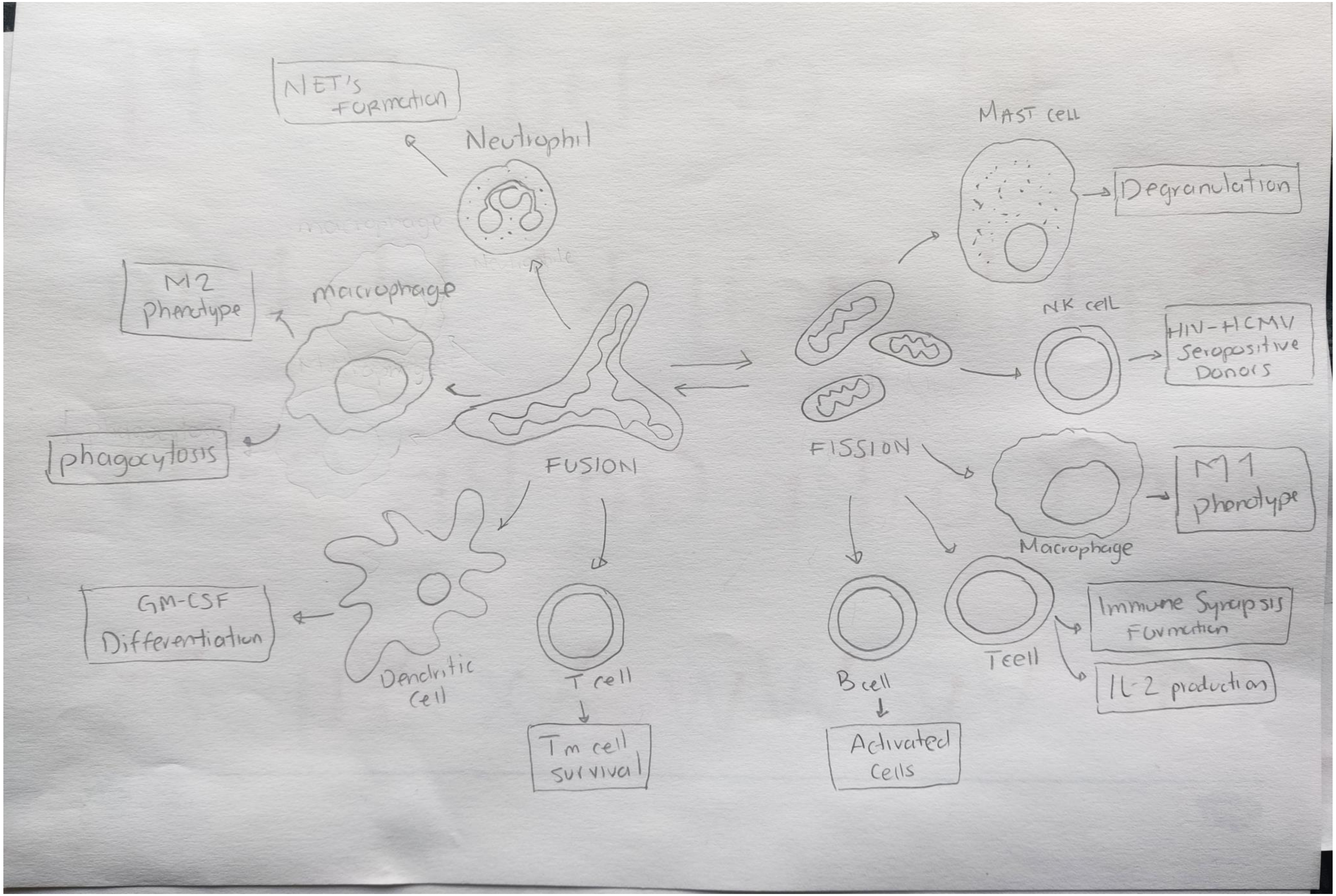

NET's Formation
Neutrophil
Mast cell
Degranulation
M2 phenotype
macrophage
NK cell
HIV-HCMV Seropositive Donors
phagocytosis
FUSION
FISSION
M1 phenotype
Macrophage
GM-CSF Differentiation
Dendritic cell
T cell
B cell
Tcell
Immune Synapsis Formation
IL-2 production
Tm cell survival
Activated Cells

Figure 3

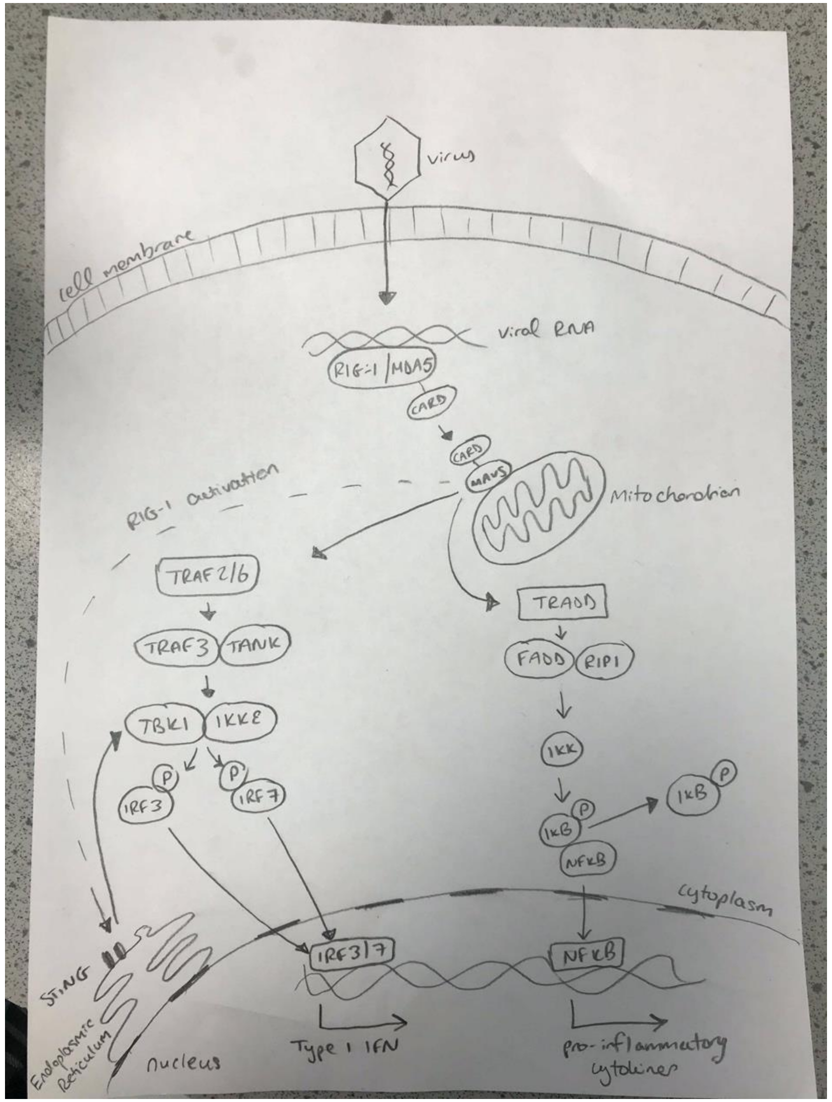
virus
cell membrane
Viral RNA
RIG-1/MDA5
CARD
CARD
MAVS
Mitochondrion
RIG-1 activation
TRAF2/6
TRAF3
TANK
TBK1
IKKε
P
P
IRF3
IRF7
TRADD
FADD
RIP1
IKK
IκB
P
IκB
P
NFκB
cytoplasm
STING
Endoplasmic Reticulum
IRF3/7
NFκB
Type 1 IFN
pro-inflammatory cytokines
nucleus

Figure 4

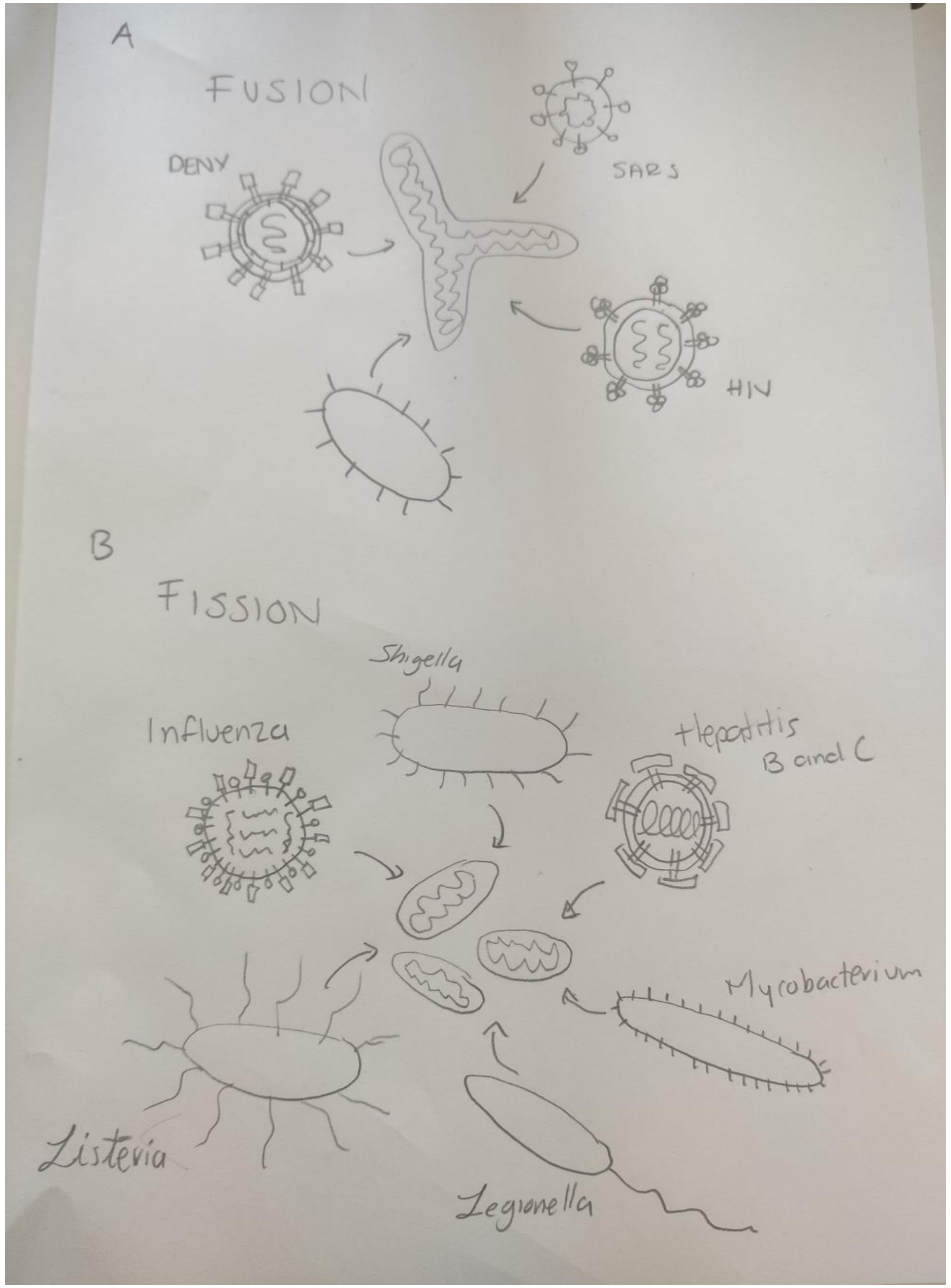
A
FUSION
DENV
SARS
HIV
B
FISSION
Shigella
Influenza
Hepatitis B and C
Mycobacterium
Listeria
Legionella

Table 1

Compound	Promotes	Action	Functional effect	Reference
Mito-C	Fission	Recruitment of DRP1	• Antiviral effect<br>• Inhibits Dengue virus replication<br>• Inhibits IAV replication	Molino *et al*, 2020; Pila-Castellanos *et al*, 2021.
Mdivi-1	Fusion	DRP1 Inhibitor	• Alleviates kidney damage in a model of cell sepsis-induced acute kidney injury.<br>• Prevents degranulation of mast cells in allergies and atopic dermatitis	Liu *et al*, 2020. Zhang *et al*, 2011.
P110	Fusion	DRP1 Inhibitor	• Protective against in vitro and in vivo models of septic cardiomyopathy	Haileselassie *et al*, 2019.
MitoQ	Fusion	Antioxidant	• Protective against 6-OHDA-induced mitochondrial fission	Solesio ME *et al*, 2013.
Melatonin	Fusion	Antioxidant	• Potential adjuvant in COVID-19 vaccines to enhance mitochondrial quality	Zhang R *et al*, 2020.
BGP-15	Fusion	Hydroxylamine derivative	• Protects lung structure in a pulmonary arterial hypertension in vivo model	Szabo A *et al*, 2018.
6-phenylhexanamide derivative mitofusin activators	Fusion	MFN activator	• Induces fusion in neurons<br>• Protective against CMT2A	Dang X *et al*, 2020.
